# A Simple MRI Score Predicts Pathological General Movements in Very Preterm Infants with Brain Injury—Retrospective Cohort Study

**DOI:** 10.3390/children11091067

**Published:** 2024-08-30

**Authors:** Monia Vanessa Dewan, Pia Deborah Weber, Ursula Felderhoff-Mueser, Britta Maria Huening, Anne-Kathrin Dathe

**Affiliations:** 1 Neonatology, Paediatric Intensive Care and Paediatric Neurology, Department of Paediatrics I, University Hospital Essen, University of Duisburg-Essen, 45122 Essen, Germany; monia.dewan@uk-essen.de (M.V.D.); ursula.felderhoff@uk-essen.de (U.F.-M.); britta.huening@uk-essen.de (B.M.H.); 2 Centre for Translational Neuro- and Behavioural Sciences, C-TNBS, Faculty of Medicine, University of Duisburg-Essen, 45122 Essen, Germany; 3 Department of Health and Nursing, Occupational Therapy, Ernst-Abbe-University of Applied Sciences, 07745 Jena, Germany

**Keywords:** cerebral magnetic resonance imaging, scoring system, brain injury, preterm infant, general movements, fidgety movements

## Abstract

Background/Objectives: Very preterm infants are at increased risk of brain injury and impaired brain development. The Total Abnormality Score and biometric parameters, such as biparietal width, interhemispheric distance and transcerebellar diameter, are simple measures to evaluate brain injury, development and growth using cerebral magnetic resonance imaging data at term-equivalent age. The aim of this study was to evaluate the association between the Total Abnormality Score and biometric parameters with general movements in very preterm infants with brain injury. Methods: This single-center retrospective cohort study included 70 very preterm infants (≤32 weeks’ gestation and/or <1500 g birth weight) born between January 2017 and June 2021 in a level-three neonatal intensive care unit with brain injury—identified using cerebral magnetic resonance imaging data at term-equivalent age. General movements analysis was carried out at corrected age of 8–16 weeks. Binary logistic regression and Spearman correlation were used to examine the associations between the Total Abnormality Score and biometric parameters with general movements. Results: There was a significant association between the Total Abnormality Score and the absence of fidgety movements [OR: 1.19, 95% CI = 1.38–1.03] as well as a significant association between the transcerebellar diameter and fidgety movements (Spearman ρ = −0.269, *p* < 0.05). Conclusions: Among very preterm infants with brain injury, the Total Abnormality Score can be used to predict the absence of fidgety movements and may be an easily accessible tool for identifying high-risk very preterm infants and planning early interventions accordingly.

## 1. Introduction

Very preterm infants (VPTs, infants born at ≤32 weeks gestation) are at increased risk of adverse neurodevelopmental outcomes later in life and every domain of neurodevelopment (e.g., cognitive, motor, sensory or socio-emotional) might be affected [1,2,3]. Concerning motor development, up to 50% of VPT suffer from minor impairments such as developmental coordination disorders [4,5]. A dreaded consequence of prematurity—despite declining rates—is cerebral palsy (CP), which affects 7–10% of VPTs [6,7,8,9]. CP comprises a group of permanent disorders of movement and posture, which are caused by non-progressive damages to the brain during the fetal or infant development [10].

Preterm birth occurs in a period of enormous brain growth and maturation, making the immature brain susceptible to brain injury and dysmaturation [11,12]. The perinatal period to around the second year of a child’s life represents a significant period of brain development and neuroplasticity, which might offer a time window for rehabilitative and therapeutic interventions [13]. Thus, early identification of high-risk infants of severe disabilities are crucial for supporting their future development [14].

Cerebral magnetic resonance imaging (cMRI) studies, usually performed at term-equivalent age (TEA), give valuable insights into the pattern of brain injuries and maturational abnormalities caused by prematurity. Around 25–30% of VPTs show some, mostly mild to moderate, forms of brain injury in cMRI at TEA [15,16]. Patterns characteristic of brain injury in VPTs are intraventricular hemorrhages (IVHs), white matter injuries (WMIs) and cerebellar hemorrhages (CBHs). Signs of brain dysmaturation include the delay of gyration and myelination as well as impaired brain growth of both grey and white matter [17,18,19]. 

For a structured evaluation of brain injuries and/or developmental and growth abnormalities in cMRI at TEA, different scoring systems have been developed [18,20,21]. However, these scores are mostly too time consuming (e.g., by including methods such as volumetry) for clinical routine and/or do not account for all dimensions of brain alterations seen in VPTs (including brain injury, dysmaturation and impaired growth) The previously published Total Abnormality Score (TAS) complemented with biometric parameters (biparietal width (BPW), interhemispheric distance (IHD), transcerebellar diameter (TCD)) was identified as a simple approach to evaluate cMRI of VPTs at TEA. The TAS consists of an injury score (subcategories IVHs, WMIs, CBHs) and a developmental score (subcategories gyration, myelination, ventricular dilatation (VD)) [22,23]. The novelty of the TAS is that it is a separate evaluation of brain injury and development. In contrast to other scores, brain injury is assessed separately for both hemispheres [23]. The structured evaluation using the TAS and biometric parameters to study the relationship between brain injury, development and growth among VPTs with later neurodevelopmental outcomes has significant clinical value. If the TAS and biometric parameters are predictive of later neurodevelopmental outcomes, clinicians can use them as easily accessible tools of early identification of high-risk infants, counsel parents and introduce rehabilitative and therapeutic interventions as early as at TEA. 

The analysis of general movements (GMs) by Prechtl has a high predictive value for later severe motor impairment, such as CP [24]. GMs are a spontaneous pattern of movements that first appear intra-uterine at a gestational age (GA) of around 9–10 weeks [25]. The pattern of movements changes sequentially and provides insight into the integrity of the maturing central nervous system. Within the corrected age of 3–5 months, fidgety movements (FMs) are the predominant pattern of GMs in an awake infant [26]. The absence of FMs is highly predictive for the development of CP in later life with a sensitivity of 98% and specificity of 95% [27,28]. The TAS has not yet been investigated for its possible association with absent FMs as an early marker for CP.

The primary objective of this study was to examine the association between the TAS and the TAS subcategories brain injury score and developmental score at TEA with GMs at the corrected age of 8–16 weeks in VPTs with brain injury. If there was an observed association between the TAS and GMs, the secondary objective was to examine the associations between each of the items of the TAS, biometric parameters and GMs.

We hypothesized that (1a) the TAS and (1b) the TAS subcategories brain injury score and developmental score will be associated with the absence of FMs in VPTs with brain injury. (2) We also hypothesized that the TAS items and biometric parameters will be associated with the absence of FMs among this population.

## 2. Materials and Methods

### 2.1. Patients

Infants born and/or treated in a level-three neonatal intensive care unit (NICU) between January 2017 and June 2021 with a GA ≤ 32 weeks and/or a birth weight < 1500 g were included. These infants were included because they are at high risk of later developmental impairments and routinely undergo cMRI scan at TEA, as well as GM analysis at the corrected age of 8–16 weeks [17,29]. Infants with malformations, chromosomal anomalies or connatal infections affecting brain development were excluded from this study. Furthermore, only infants with brain injury diagnosed in cMRI at TEA (TAS > 0) were included in this study because they were considered at high risk of absent FM, which was the primary outcome of this study. Clinical information on peri- and postnatal characteristics was obtained from hospital records. 

This study was performed according to the principles of the Declaration of Helsinki and approved by the Ethics Committee of University of Duisburg-Essen (18-8388-BO, 30 August 2018).

### 2.2. cMRI Process 

Data for TAS, TAS subcategories brain injury score and developmental score, and biometric parameters (BPW, IHD, TCD) were obtained from cMRI. As part of routine care for VPTs, cMRI was performed on a 3 Tesla MRI scanner (Skyra, Siemens Healthcare, Erlangen, Germany). Infants were examined in an MRI-compatible incubator (Lammers Medical Technology nomag IC, Luebeck, Germany). A dedicated 8-channel neonatal head coil was used. Before imaging, infants were swaddled and fed. An individual noise protection was adapted with a malleable paste (Affinis^®^) and mini muffs (Natus Medical Incorporated, Seattle, WA, USA) to reduce noise. During the examination, the infants’ heart rate and oxygen saturation were monitored with an MRI-compatible pulse oxymeter (Invivo Essential, Orlando, FL, USA). Patients were accompanied by instructed pediatricians and nurses. Chloral hydrate (25–50 mg/kg body weight) was applied through a gastric tube to avoid motion artefacts only if necessary. Details on the standard imaging protocol have been previously published [23]. It included transversal T2-weighted turbo spin echo, T1-weighted 3D fast low-angle shot (FLASH) and susceptibility-weighted (SWI) and diffusion-weighted (gradients: b0, b700, b1000) imaging. The duration of a cMRI scan was around 20 min.

### 2.3. cMRI Analysis: TAS and Brain Biometric Parameters 

All cMRI data were assessed by experienced pediatric radiologists or neuroradiologists as part of our routine care. Blinded to the clinical course and the radiological reports, two investigators (neonatologist/pediatrician; B.M.H./M.V.D.) experienced with the application of the TAS [22,23] scored the cMRI data using the criteria listed in Table A1 in Appendix A. The TAS results were compared to the radiological reports. In cases of discordance, results were discussed with an experienced pediatric radiologist. Additionally, the biometric parameters BPW, IHD and TCD were measured using the cMRI data as previously described [23]. A BPW z score of <−0.5 defined a small BPW and an IHD ≥4.0 mm was defined as enlarged among the VPT [15]. An impaired cerebellar growth was defined as TCD < 50 mm [30].

### 2.4. General Movement Analysis

To assess the absence of FMs, GMs were assessed during the fidgety period at a corrected age of 8–16 weeks using the methodology of Prechtl [24,29,31]. The GMs were judged by two advanced certified and experienced raters (B.M.H, A.-K.D.). A third rater was involved if there was a disagreement between these two primary raters. The movements were classified as normal, abnormal or absent FMs according to the global character of the movement as part of routine care. FMs are small movements of moderate speed and variable acceleration. These occur in neck, trunk and limbs in all directions. FMs can be observed continually in an awake infant who is not crying or sucking. Abnormal FMs are of larger amplitude, and the speed and jerkiness are moderately exaggerated. These movements are rare. Absence of FMs means that FMs are not or not continuously observed [24].

### 2.5. Statistics

SPSS 29.0 (IBM SPSS Statistics for Windows, IBM Corp., NY, USA) was used for data analysis. Descriptive statistics were used to examine the demographic and clinical characteristics of the participants, using absolute and relative frequencies or median and interquartile range (IQR). 

For the primary objective, (1a) a binary logistic regression was conducted to examine the association between the TAS at TEA and the absence of FM, after controlling for the child’s sex and GA. (1b) This analysis was repeated with the TAS subcategories brain injury score and developmental score to examine the associations between these subcategories’ scores and the absence of FMs.

For the secondary objective (2), Spearman Correlation was used to examine the associations between each of the items of the TAS, biometric parameters and the absence of FMs.

Sample size was determined using recommended guidelines for logistic regression.

A minimum of ten outcome events per predictor variable (EPV) is recommended, which required [32,33,34,35] a minimum sample size of 30 for the three predictors in the regression model for primary hypothesis 1a and 40 for 1b. Therefore, 70 VPTs was the targeted sample size when beginning this retrospective study. The EPV was used as the minimum sample size criterion, as there are no studies to date on the prediction of FMs by the TAS. Studies on the association between a structured MRI score, e.g., the Kidokoro score, in VPTs at TEA and FMs are rare [36,37]. In a study by Harpster et al. [36], 375 infants were examined, 121 (32.3\%) infants had brain abnormalities and 17 infants had no FMs. In a study by Wang et al. [37], 8 infants with CP and 8 without CP were analyzed to examine the association between a structured MRI score with FM. The existing body of evidence does not provide sufficient information to inform the sample size calculation for this study—thus, the sample size calculation recommended for binary logistic regression was adopted.

## 3. Results 

### 3.1. Patients

**Figure 1:**
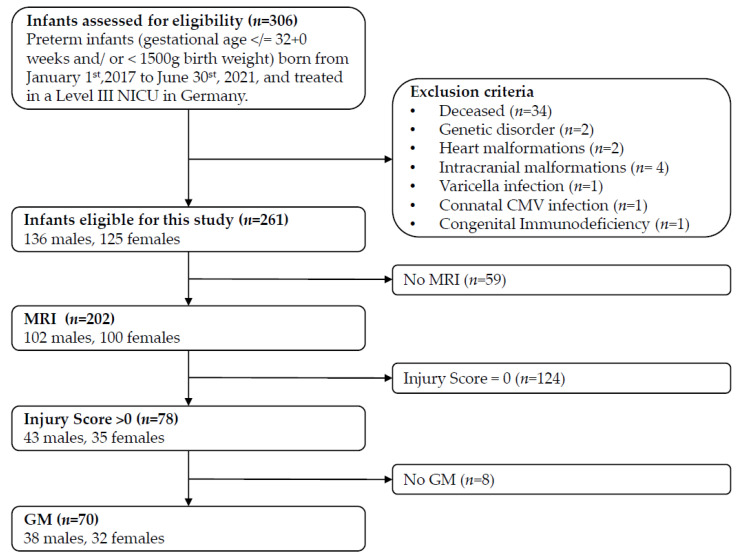
Patient flow and exclusion criteria. Notes. NICU = neonatal intensive care unit; CMV = cytomegaly virus; MRI = magnetic resonance imaging; GM = general movement.

From 1 January 2017, to 30 June 2021, 306 VPTs with ≤32 + 0 weeks gestation and/or <1500 g birth weight were born in a level-three NICU. Among these infants, 261 VPTs (85.3%) were eligible for this retrospective study. A cMRI at TEA was performed in 202 (77.0%) VPTs with 78 (38.6%) showing any grade of brain injury. The final cohort comprised of 70 VPTs, as eight VPTs had no GM analysis. Figure 1 shows the patient flow that determined the final sample size for this study.

### 3.2. Cohort Characteristics

Among the 70 VPTs included in this study, 32 (45.7%) were female. Median GA at birth was 29 weeks [IQR 25.6–30.6] and median birth weight was 1068 g [IQR 710–1466]. Table 1 summarizes the peri- and postnatal characteristics of the study sample. 

### 3.3. Descriptive Analyses of cMRI and GM Data

The cMRI at TEA was performed at median 40 weeks [IQR 40.0–40.1] (Table 2). The median TAS was 7 [IQR 4–10] (range 3–25). Median Injury Score was 4 [IQR 2–8], while median Developmental Score was 2 [IQR 2–3]. IVH in cMRI was found in 38 (54.3%), CBH in 28 (40.0%) and WMI in 34 (48.6%) VPTs. For brain biometric parameters, there was a median BPW of 77.4 mm [IQR 73.4–81.5] with a small BPW in 19 (28.4%) VPTs. Median IHD was 2.2 [1.8–3.3] with 15 (22.7%) VPTs having an enlarged IHD. Median TCD was 52.25 mm [49.1–55.3] with 20 (28.6%) VPTs showing impaired cerebellar growth (TCD < 50 mm). Corrected median age at GM analysis was 13 weeks [IQR 12–13]. Among the 70 VPTs analyzed, 6 (8.6%) showed no FMs; no infants showed abnormal FMs. 

### 3.4. Association between TAS and the Absence of Fidgety Movements at the Corrected Age of 3 Months

After controlling for the child’s sex and GA, there was a significant association between the TAS and the absence of FM (odds ratio: 1.19 [95% CI = 1.38–1.03] (Table 3)). For a point increase in the TAS, there was a lower likelihood of FM. 

After controlling for the child’s sex and GA, there were no significant associations between the TAS subcategories brain injury score and developmental score and the absence of FMs. 

### 3.5. Associations between Cerebellar Injury and Growth as Well as the Grade of Ventricular Dilatation with the Absence of Fidgety Movements 

There were significant associations between several TAS items and a biometric parameter with FMs, including the CBH grade (Spearman ρ = −0.282, *p* = 0.018, *n* = 70), VD grade (ρ = −0.333, *p* = 0.004, *n* = 70) and TCD (Spearman ρ = 0.269, *p* = 0.018, *n* = 67) (Table 4).

The explorative analysis showed significant associations between several TAS items and biometric parameters, including CBH with WMI and TCD; Grade of gyration with myelination and VD; VD with IVH, myelination, gyration and TCD; TCD with IVH, CBH, VD and BPW.

## 4. Discussion

The aim of this study was to evaluate the association between the TAS and biometric parameters on cMRI at TEA with GMs at the corrected age of 8–16 weeks in VPTs with brain injury. The results show a significant association between the TAS and the absence of FMs in VPTs with brain injury on cMRI at TEA. There were no significant associations between the TAS subcategories brain injury score and developmental score and the absence of FMs. Among the TAS items, there was a significant association only between the CBH and VD grades with the absence of FMs. Among the biometric parameters, there was a significant association only between TCD and FMs. 

Different MRI scores have been applied to predict GM at 3 months corrected age and to identify structural abnormalities and brain pathologies underlying the development of early adverse motor outcomes, with different results. A recent study by Harpster et al. showed that the Kidokoro global abnormality score correlates with the absence of FMs at 3 months corrected age. In addition to the Kidokoro score, IVH and several biometric parameters (pons, cerebellar vermis, frontal extra-axial space) were assessed, among which an increased thickness of the pons decreased and an increased extra-axial space increased the odds of absent FMs [36]. The same score was used by Wang et al. who found severe white matter injury strongly associated with adverse motor development and later diagnosis of CP [37]. Maeda et al. also used the Kidokoro Score, which was associated with the quality of GMs but at preterm and term-equivalent age. In that study, both preterm and term GMs were associated with cerebellar abnormalities [39]. Spittle et al. used a standardized scoring system to grade white and grey matter pathology. In that study, cerebral diffuse white matter abnormalities, IVH and periventricular leukomalacia (PVL), but not grey matter abnormalities at TEA were associated with pathological GM at 3 months corrected age. The study group concluded that WMI was the major contributor to adverse early motor development [40]. In a more recent study, the same study group applied six different biometric parameters (bifrontal, biparietal and transverse cerebellar diameters, IHD and diameter of left and right ventricles) in addition to the white and grey matter scoring system to investigate the association between impaired brain growth and abnormal GMs. Consistent with our findings, a reduced TCD was the only predictive biometric parameter of abnormal GMs independent of WMI and high-grade IVH [41]. 

Cerebellar underdevelopment is commonly seen in VPTs and might last until adolescence and beyond [42,43]. In our study, a reduced TCD was observed in 28.6% of VPTs infants with brain injuries. The cerebellum undergoes an unparalleled development with a 4–5-fold increase in volume and 30-fold in surface between 24 and 40 weeks’ gestation, thus marking a highly vulnerable period of neurodevelopment [44,45]. Besides the association of reduced TCD and pathological GM outcome, impaired motor development at the corrected age of 24 months in VPTs with cerebellar underdevelopment including CP have been described [41,46]. Studies in older children and adolescents showed that small cerebellar volumes are associated with impaired non-motor functions such as cognition, language and behavior [47,48,49,50]. Besides the TCD, the CBH was also significantly associated with GM outcome in this study. Former studies have reported the association between CBH and later motor impairment including CP. In children with isolated CBH, long-term cognitive, learning and behavioral dysfunction are also known [51]. 

Although high-grade IVH and PVL as leading pathologies underlying the development of GMs and later CP have been described by several studies [52,53], the results of this study further suggest the clinical importance of cerebellar injury and growth for early motor development. They also support the use of routine cMRI at TEA in VPTs, which remains controversial because of its relatively high costs compared to the benefits [54]. However, MRI is superior to CUS to detect CBH. While in MRI studies up to 19–37% of VPTs are diagnosed with CBH, only 7–9% show CBH in cerebral ultrasound, underlining the importance of MRI based evaluation of cerebellar injury. cMRI is also superior in detecting other small injuries in the white matter and impairments of growth and maturation [17]. 

### 4.1. Limitations

The study results are limited by the relatively small final cohort size of 70 participants. In 59 (22.6%) infants, no cMRI was available due to parental rejection and/or reduced MRI capacity during the COVID-19 pandemic. The cohort size was further reduced because only infants with brain injury were evaluated as they are at high-risk for severe motor outcome reflected by the absence of FMs, which was the primary outcome of this study. Thus, the study results are limited by exclusion of infants with injury score = 0, with a possible effect on the results. Nevertheless, the study sample is clinically relevant and consistent with the recommendations of the American Academy of Pediatrics, where routine cMRI is not recommended in every VPTs. Only infants with a risk factor, e.g., brain injury, are recommended to undergo a cMRI scan [55].

In our study, the number of infants without FMs at the corrected age of 8–16 weeks was low with *n* = 6 (8.6%) infants, but within the expected range according to other studies [56,57]. It is known that the prevalence of children without FMs is low. Logistic regression analysis is susceptible of variables with few events compared to the sample size [58]. Logistic regression models with less than 100 participants may overestimate the effect [59]. This bias is known as sparse data or small sample bias and occurs when the event rate is relatively low compared to the sample size [58]. Nevertheless, small sample sizes are a common problem in clinical studies and larger studies are necessary to confirm the results of this study. Another limitation of the regression model is that it only controlled for child sex and GA. Our sample was too small to include intervention effects as a confounder in the model. It is difficult to reliably record retrospective therapies in terms of frequency, duration and method. Larger prospective studies could attempt this in the future. Due to the cohort´s age, only short-term motor outcome was assessed with the GM analysis. In a former study the prognostic value of the TAS and biometric parameters for cognitive and motor outcome at the corrected age of 24 months tested with the Bayley-Scales of infant development II was low [23]. The TAS is perhaps better suited for predicting severe motor impairment than motor and cognitive developmental scores. The predictive value of the TAS for CP should be investigated in the future as well as the role of cerebellar injury and growth in the development of severe motor impairment.

### 4.2. Strengths

The key strength of this study is the evaluation of the TAS based on high-quality cMRI including highly blood sensitive SWI sequences, which facilitates the detection of smallest hemorrhagic lesions. The evaluation of the cMRI scans was performed by an experienced team consisting of pediatric radiologists/neuroradiologists and two pediatricians/neonatologists blinded for clinical course. Another strength of this study is the identification of a simple tool for early detection of future neurodevelopmental impairments. With the TAS and biometric parameters, a simple tool for the overall evaluation of VPTs’ brain injury, development and growth were used which can fit into routine clinical practice and may be used as a complementary method to identify high-risk VPTs at TEA. In our cohort, the absence of FMs was predicted by the TAS, whereas the prediction with the TAS subcategories injury score and developmental score was not significant. This fact underlines the importance of evaluating all dimensions of brain abnormalities, which is the strength of the TAS. 

## 5. Conclusions

The TAS is a simple scoring system for a structured evaluation of cMRI of VPTs at TEA. The TAS supplemented by brain biometrics give an overall view on brain injury, development and growth. Its application is not time consuming and thus suitable for routine clinical practice. In this study, the TAS was predictive of the absence of FMs at the corrected age of 8–16 weeks in VPTs with brain injury. As the absence of FMs is highly predictive for the development of CP, future studies need to evaluate the predictive value of the TAS for this severe movement disorder. Furthermore, results of this study suggest the important role of cerebellar injury and growth in the development of FMs, which must also be elucidated in future studies.

## Figures and Tables

**Table 1:** Peri- and postnatal characteristics.

	Cohort (*n* = 70)
Female, *n* (%)	32 (45.7)
Gestational age at birth in weeks, median [IQR]	29 [25.79–30.57]
Weight at birth in grams, median [IQR]	1068 [710–1466]
SGA, *n* (%)	14 (20)
Length at birth in centimeters, median [IQR]	36 [31–40]
Head circumference at birth in centimeters, median [IQR]	25.75 [22.88–28.50]
Inborn, yes, *n* (%)	64 (91.4)
Caesarean section, yes, *n* (%)	60 (85.7)
5-min APGAR score, median [IQR]	8 [6–9]
10-min APGAR score, median [IQR]	9 [7–9]
Umbilical cord pH, median [IQR]	7.33 [7.23–7.37] ^a^
Antenatal betamethasone (2 applications at intervals of 24 h), yes, *n* (%)	42 (71.2) ^b^
Parental ISCED maximal, median [IQR]	4 [3–6] ^c^
FIP, yes, *n* (%)	3 (4.3)
NEC, yes, *n* (%)	3 (4.3)
Operations in general, yes, *n* (%)	22 (31.4)
Postnatal sepsis, yes, *n* (%)	32 (45.7)
BPD, yes, *n* (%)	20 (28.6)
RDS, yes, *n* (%)	67 (100) ^d^
Surfactant, yes, *n* (%)	46 (69.7) ^e^
Pneumothorax, yes, *n* (%)	6 (8.7) ^f^
Neonatal seizure, yes, *n* (%)	8 (11.4)
Catecholamines, yes, *n* (%)	16 (22.9)
PDA, yes, *n* (%)	47 (67.1)
Treated PDA, yes, *n* (%)	28 (40)
ROP, yes, *n* (%)	36 (51.4)
Treated ROP, yes, *n* (%)	7 (11.3) ^g^

Notes: IQR = interquartile range; SGA = small for gestational age with weight of birth < 10th percentile; ISCED = International Standard Classification of Education. FIP = focal intestinal perforation; NEC = necrotizing enterocolitis; BPD = bronchopulmonary dysplasia; RDS = respiratory distress syndrome; PDA = patent ductus arteriosus, Treated PDA = pharmacologic and/or surgical treatment; ROP = retinopathy of prematurity; Treated ROP = pharmacologic and/or surgical treatment. Descriptive statistics are based on ^a^ *n* = 66, ^b^ *n* = 59, ^c^ *n* = 63, ^d^ *n* = 67, ^e^ *n* = 66, ^f^ *n* = 69, ^g^ *n* = 62; otherwise *n* = 70.

**Table 2:** cMRI data at term and motor outcome at 3 months corrected age.

	Cohort (*n* = 70)
Gestational age at cMRI, weeks, median [IQR]	40 [40.00–40.14]
Head circumference in centimetres at cMRI, median [IQR]	34 [33.00–35.25] ^a^
TAS, median [IQR]	7 [4.00–10.25]
Injury Score, median [IQR]	4 [2–8]
IVH (Score), yes, *n* (%)	38 (54.3)
IVH grade I, yes, *n* (%)	2 (2.9)
IVH grade II, yes, *n* (%)	21 (30.0)
IVH grade III, yes, *n* (%)	1 (1.4)
IVH grade IV, yes, *n* (%)	14 (20.0)
CBH (Score), yes, *n* (%)	28 (40.0)
CBH grade I, yes, *n* (%)	10 (14.3)
CBH grade II, yes, *n* (%)	7 (10.0)
CBH grade III, yes, *n* (%)	11 (15.7)
White matter injury (Score), yes, *n* (%)	34 (48.6)
WMI grade I, yes, *n* (%)	11 (15.7)
WMI grade II, yes, *n* (%)	7 (15.7)
WMI grade III, yes, *n* (%)	1 (10.0)
WMI grade IV, yes, *n* (%)	15 (21.4)
Development Score, median [IQR]	2 [2–3]
Myelination, median [IQR]	0 [0-0]
Gyration, median [IQR]	1 [1-1]
VD, median [IQR]	1 [1-1]
Metric parameters	
BPW, mm, median [IQR]	77.4 [73.4–81.5] ^b^
BPW z-score less than -0.5, *n* (%)	19 (28.4) ^b^
IHD, mm, median [IQR]	2.2 [1.8–3.3] ^c^
IHD ≥ 4 mm, *n* (%)	15 (22.7) ^c^
TCD, mm, median [IQR]	52.25 [49.08–55.3] ^c^
TCD growth impairment (<50 mm), *n* (%)	20 (28.6) ^c^
Corrected age at GMA, weeks, median [IQR]	13 [12–13]
Absence of fidgety movements, *n* (%)	6 (8.6)

Notes. IQR = interquartile range; IVH = intraventricular hemorrhage; CBH = cerebellar hemorrhage; PVL = periventricular leukomalacia; TAS = Total Abnormality Score; VD = ventricular dilatation; BPW = biparietal width; BPW z-score based on the cohort of very preterm infants with BPW values (n = 67); IHD = interhemispheric distance; TCD = transcerebellar diameter; GMA = General movements assessment. Descriptive statistics are based on ^a^ *n* = 65, ^b^ *n* = 67, ^c^ *n* = 66,; otherwise *n* = 70.

**Table 3:** Regression analysis investigating effects of TAS, child sex and gestational age on fidgety movements (*n* = 70).

	B	*p*	Odds Ratio	95% CI
Step 1				
TAS	−0.184	0.011	0.832	0.721–0.959
Total R^2^		0.211 **		
Step 2				
TAS	−0.168	0.025	0.846	0.730–0.979
Child sex	−0.884	0.375	0.413	0.059–2.907
Gestational age	0.196	0.293	1.216	0.844–1.752
Total R^2^		0.258 *		

Notes. The binary logistic regression model was statistically significant with a small effect, χ2(3) = 8.47, *p* = 0.04, Nagelkerke’s R^2^ = 0.26 [38]. CI = confidence interval; TAS = Total Abnormality Score, R^2^ = Nagelkerke’s R^2^. * *p* < 0.05, ** *p* < 0.01.

**Table 4:** Spearman Correlation of TAS items, biometric parameters and the absence of fidgety movements.

	FM	IVH	CBH	WMI	Myelination	Gyration	VD	BPW	IHD
Injury score									
IVH grade	−0.130								
CBH grade	−0.282 *	0.144							
WMI	−0.053	−0.118	−0.262 *						
Developmental score									
Myelination grade	−0.194	0.112	0.011	0.146					
Gyration grade	−0.209	0.213	0.084	−0.019	0.559 **				
VD grade	−0.333 **	0.534 **	−0.016	−0.072	0.247 *	0.296 *			
Metric data									
BPW	0.129	−0.210	−0.060	−0.093	−0.185	−0.018	−0.057		
IHD	0.019	0.120	−0.039	−0.016	−0.226	−0.063	0.088	0.193	
TCD	0.269 *	0.423 **	−0.282 *	−0.041	0.157	−0.191	−0.354 **	0.513 **	0.222

Notes. FM = fidgety movement; IVH = intraventricular hemorrhage; CBH =cerebellar hemorrhage; WMI = grade of white matter injury; VD = ventricular dilatation; BPW = biparietal width; IHD = interhemispheric distance; TCD = transcerebellar diameter. * *p* < 0.05, ** *p* < 0.01. The sample size varied depending on the pair of variables (*n* = 64–70).

## Data Availability

The dataset used and/or analyzed for the study is available from the corresponding author upon reasonable request.

## Appendix A

**Table A1:** The Total Abnormality Score [22,23].

Injury Score ^a^	Developmental Score ^a^
**IVH (grades 0–4), (T1, T2, SWI)** 0 = no hemosiderin deposits 1 = hemosiderin deposits or post hemorrhagic cysts within caudo-thalamic notch 2 = hemosiderin deposits outside notch, along ventricle wall without VD 3 = obvious VD with evidence of previous ventricular hemorrhage 4 = parenchymal hemorrhagic lesions > 3 mm or posthemorrhagic cystic encephalopathy	**Myelination (grades 0–4), (T1)** 0 = ≥1/3 PLIC + centrum semiovale 1 = ≥1/3 PLIC + optic radiation, not centrum semiovale 2 = ≥1/3 PLIC, but not in centrum semiovale and optic radiation 3 = ≤1/3 PLIC, clearly visible 4 = no myelin in PLIC
**White matter lesions (grades 0–4), (T1, T2)** 0 = no signs for lesions in peri-ventricular WM on T1 and T2 1 = punctate lesions ≤ 3 mm and <3 lesions 2 = punctate lesions ≤ 3 mm in corticospinal tract or punctate lesions ≤ 3 mm and ≥3 lesions in <3 regions (frontal, temporal, parietal, occipital) 3 = extensive lesions (>3 mm and/or >3 lesions in ≥3 regions) 4 = cystic lesions in periventricular WM or cluster (≥6 lesions)	**Gyration (grades 0–2), (T1)** 0 = 40 wks, tertiary gyri/sulci inferior temporal and inferior occipital 1 = 36–38 wks, additional secondary gyri/sulci transversi and inferior temporal 2 = 34–36 wks, present marginal sulcus, paracentral gyrus, secondary gyri front.
**CBH (grades 0–3), (T1, T2, SWI)** 0 = no hemorrhage 1 = hemorrhage ≤ 3 mm and ≤3 lesions 2 = punctate lesion(s) > 3 mm and/or >3 lesions per hemisphere 3 = extensive lesions	**Ventricular dilatation (VD), (grades 0–4), (T2)** 0 = slim ventricles both sides 1 = mild dilatation, but clear concave shape, with modelling to gyri 2 = moderate VD, borderline shape 3 = severe VD, rounded ventricular horns, convex shape 4 = porencephalie

Notes. TAS = Total Abnormality Score; PVL = periventricular leukomalacia; WM = white matter; IVH = intraventricular hemorrhage; VD = ventricular dilatation; SWI = susceptibility-weighted imaging, CBH = cerebellar hemorrhage; PLIC = posterior limb of internal capsule; wks = weeks, DGMA = deep grey matter area. ^a^ Injury and Developmental Scores are summed up to TAS. Note that the Injury Score is obtained separately for the right and left hemisphere.
